# Axillary mechanical circulatory support improves renal function prior to heart transplantation in patients with chronic kidney disease

**DOI:** 10.1038/s41598-023-46901-7

**Published:** 2023-11-11

**Authors:** Ji-Min Jang, Tambi Jarmi, Basar Sareyyupoglu, Jose Nativi, Parag C. Patel, Juan C. Leoni, Kevin Landolfo, Si Pham, Daniel S. Yip, Rohan M. Goswami

**Affiliations:** 1 Division of Heart Failure and Transplant, Mayo Clinic Florida, 4500 San Pablo Road, Jacksonville, FL 32246 USA; 2 Division of Transplant Nephrology, Mayo Clinic Florida, Jacksonville, USA; 3 Department of Cardiothoracic Surgery, Mayo Clinic Florida, Jacksonville, USA

**Keywords:** Cardiology, Medical research, Nephrology

## Abstract

Impaired kidney function is often associated with acute decompensation of chronic heart failure and portends a poor prognosis. Unfortunately, current data have demonstrated worse survival in patients with acute kidney injury than in patients with chronic kidney disease during durable LVAD placement as bridge therapy. Furthermore, end-stage heart failure patients undergoing combined heart-kidney transplantation have poorer short- and long-term survival than heart transplants alone. We evaluated the kidney function recovery in our heart failure population awaiting heart transplantation at our institution, supported by temporary Mechanical Circulatory Support (tMCS) with Impella 5.5. The protocol (#22004000) was approved by the Mayo Clinic institutional review board, after which we performed a retrospective review of all patients with acute on chronic heart failure and kidney disease in patients considered for only heart and kidney combined organ transplant and supported by tMCS between January 2020 and February 2021. Hemodynamic and kidney function trends were recorded and analyzed before and after tMCS placement and transplantation. After placement of tMCS, we observed a trend towards improvement in creatinine, Fick cardiac index, mixed venous saturation, and glomerular filtration rate (GFR), which persisted through transplantation and discharge. The average duration of support with tMCS was 16.5 days before organ transplantation. The median pre-tMCS creatinine was 2.1 mg/dL (IQR 1.75–2.3). Median hematocrit at the time of tMCS placement was 32% (IQR 32–34), and the median estimated glomerular filtration rate was 34 mL/min/BSA (34–40). The median GFR improved to 44 mL/min/BSA (IQR 45–51), and serum creatinine improved to 1.5 mg/dL (1.5–1.8) after tMCS. Median discharge creatinine was 1.1 mg/dL (1.19–1.25) with a GFR of 72 (65–74). None of these six patients supported with tMCS required renal replacement therapy after heart transplantation. Early adoption of Impella 5.5 in this patient population resulted in renal recovery without needing renal replacement therapies or dual organ transplantation and should be further evaluated.

## Introduction

Impaired kidney function is often associated with acute decompensation of chronic heart failure and portends a poor prognosis^1^. The progressive nature of heart failure and limited survival during end stages necessitates advanced therapies. These options may include consideration for transplantation or bridge therapies. Traditional bridge-to-transplant options have included continuous inotrope therapy or implantation of durable left ventricular assist devices (LVAD). Unfortunately, current data have demonstrated worse survival in patients with acute kidney injury than in patients with chronic kidney disease during LVAD placement as bridge therapy^2^.

Based on national data from the Organ Procurement and Transplantation Network (OPTN), there have been 2561 simultaneous heart/kidney transplantations in the United States since 1988. In 2021, a total of 349 simultaneous heart/kidney transplantations were performed. Of these transplantations, it is unclear how many would have had potentially reversible kidney injury with optimization of the cardiorenal axis. In patients with New York Heart Association (NYHA) class 3–4 and American Heart Association (AHA) stage C-D heart failure, worsening kidney function often prompts the consideration to start kidney replacement therapy, a factor that is directly linked to worse outcomes in the acute setting^3, 4^. Furthermore, end-stage heart failure patients undergoing combined heart-kidney transplantation have poorer short- and long-term survival than heart transplants alone^5^.

We evaluated the kidney function recovery in a chronic heart failure population awaiting heart transplantation at our institution supported by temporary Mechanical Circulatory Support (tMCS) with Impella 5.5. Our observations showed a sustained improvement in kidney function where kidney transplantation was not required in a subset of patients with the Impella 5.5 axillary tMCS device. This has not been routinely seen with cardiogenic shock patients supported before durable LVAD, inotrope therapy, or ECMO^6–9^.

## Methods

The protocol (#22004000) was approved by the Mayo Clinic institutional review board. All methods were performed in accordance with the relevant guidelines and regulations. W**e** performed a retrospective review of all patients with acute on chronic heart failure and kidney disease in patients considered for only heart and kidney combined organ transplant and supported by Impella 5.5 between January 2020 and February 2021. Hemodynamic and kidney function trends were recorded and analyzed before and after Impella 5.5 placement and transplantation.

Our institutional protocols and multi-disciplinary approach to management has been described in previous publications and is summarized below^29^.

### Patient population

**Figure 1:**
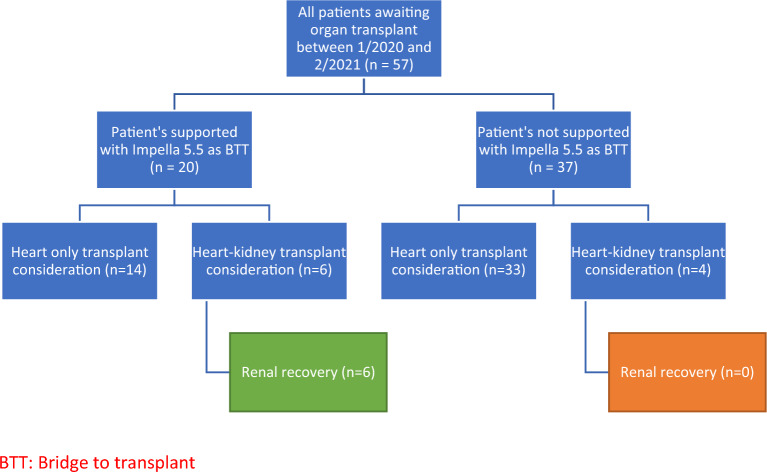
Visual representation of patients with renal recovery. BTT: Bridge to transplant.

During our review period, 57 patients underwent heart transplantation, of which 20 patients with cardiogenic shock utilized the Impella 5.5 with Smart Assist, intended as a bridge to organ transplantation (35%). The presented series of six patients were the only individuals during this timeframe who were worked up for heart and kidney transplant while supported with Impella 5.5 (Fig. 1). The kidney transplant team performed an extensive evaluation based on our institutional requirements before being considered a suitable candidate for kidney transplantation. All patients had chronic heart failure diagnosed, on average, at least 7 years before their admission for acute decompensated heart failure. Pre-admission lab values, ejection fraction, and abdominal ultrasound kidney measurements for each patient before admission (1 year prior to being admitted) are provided in Table 1 to provide a historical perspective and frame the impact of progressive cardiorenal disease in this population.

**Table 1:** Patient characteristics and data trends.

Case	1	2	3	4	5	6
Age	67	66	74	70	55	54
Sex	M	M	M	M	F	M
Etiology	NICM	Ischemic	Ischemic	NICM	NICM	NICM
BMI	28.3	29	33.9	31.25	33	29.7
Device duration, days	27	27	22	14	3	6
Survival after transplant (days)	562	547	466	291	571	485
Blood group	O	O	A	B	O	O
Diabetic	N	Y	N	N	N	N
Hemoglobin A1c (%)	5.9	8	6.2	5.7	5.8	6
LVEF pre-impella (%)	14	13	26	10	18	24
Historic lab and imaging data (1 year prior to admission)						
LVEF (%)	16	15	10	18	18	21
Creatinine g/dL	1.26	3	1.2	1.64	1.49	1.58
GFR	56	< 15	55	24	46	49
Kidney size (cm)	9.8 R / 11 L	11.5 R / 12 L	12.5 R / 12.8 L	11.7 R / 10.8 L	10.5 R / 10.5 L	12.5 R / 12.1 L
Pre-impella data (most recent data before Impella placement)						
MAP	61	68	73	90	102	73
RA	12	10	2	12	15	15
PA	54/23	40/22	72/30	63/32	75/37	45/25
PA mean	33	28	44	42	50	32
PCWP	30	14	30	35	32	25
SVO2 prior to impella (%)	35%	60%	67%	55%	52%	58%
Fick CO	4.20	2.5	5.7	3.07	2.5	3.5
Fick CI pre-impella	1.90	1.4	2.4	1.41	1.2	1.5
CPO	0.57	0.38	0.92	0.61	0.57	0.57
PAPi	2.58	1.8	21	2.58	2.5	1.33
CVP/PCWP	0.40	0.71	0.07	0.34	0.47	0.60
PVR	0.71	5.60	2.46	2.28	7.20	2.00
SVR	933	1856	996	2033	2784	1326
VIS pre-impella	10.00	7.5	4.8	2.5	2.5	8.7
Hematocrit	30.4	25.4	33.5	39.1	34.3	30.3
Pre-Impella Cr	2.11	2.37	1.64	2.63	1.6	2.08
Pre-Impella GFR	32	28	41	24	41	35
Pre-Impella CKD Stage	3	4	3	4	3	3
Pre-transplant data (most recent data before transplantation)						
MAP	73	88	76	78	72	74
SVO2 prior to transplant (%)	55%	63%	55%	67%	64%	65%
Fick CO	3.48	5.93	9.7	5.1	4.50	5.5
Fick CI	1.83	2.85	4.2	2.4	2.20	2.5
CPO	0.56	1.16	1.63	0.88	0.72	0.90
Hematocrit	29.2	26.8	31.2	38.9	36.2	29.1
Pre-TxpCr	1.92	0.72	1.5	2.3	1.36	1.45
Pre-Txp GFR	35	90	44	32	51	63
Pre-Txp CKD stage	3	1	3	3	3	2
Average LDH	388	166	253	245	315	117
VIS pre-transplant	10.00	5	3.75	2.5	2.5	5
Peri-transplant and discharge data						
UNOS status at transplant	2	2	2	2	2	1e (RV Failure)
Cardiopulmonary bypass time (min)	124	155	168	191	145	188
Cold ischemic time (minutes)	190	191	226	136	219	247
Discharge creatinine	1.1	1.05	1.3	1.6	0.96	1.1
Discharge GFR	69	74	54	42	78	75
Post-txp CKD stage	2	2	3	3	2	2
Total hospital stay (days)	49	41	42	22	15	59
Device to transplant time (days)	27	27	22	15	3	6
ICU LOS Post Txp (days)	7	5	5	4	4	3
VIS post transplant	9.12	17.3	7	11.40	7.5	8

BMI = body mass index (kg/m2), LVEF = left ventricular ejection fraction, GFR = glomerular filtration rate, MAP = mean arterial pressure, RA = right atrial pressure, PA = pulmonary artery, PCWP = pulmonary capillary wedge pressure, SVO2 = mixed venous saturation, CO = cardiac output, CI = cardiac index, CPO = cardiac power output, PAPi = pulmonary artery pulsatility index, PVR = pulmonary vascular resistance, VIS = vasoactive inotrope score, Cr= creatinine, Txp = transplant, LDH = lactate dehydrogenase, LOS = length of stay.

Here, we describe six patients who demonstrated marked kidney function improvement, eventually undergoing heart-only transplantation, who were supported with the Impella 5.5 with SmartAssist. The resultant support of patients with the Impella device likely allowed for improved hemodynamic and cardiorenal optimization—without an increased risk of hemolysis or device-related complications and avoiding renal replacement therapies in the perioperative period.

### Impella management

All patients supported with the Impella 5.5 with Smart Assist were co-managed with transplant critical care, transplant cardiology, and cardiothoracic surgery. Daily multi-disciplinary rounds were performed to provide cohesive care. For those patients being considered for heart-kidney transplantation, transplant nephrology was consulted. Our institutional practice for Impella 5.5 candidacy in the setting of progressive cardiogenic shock refractory to single or dual inotrope therapy has been previously described^29^. Progressive left ventricular failure, based on the assessment by Fick cardiac index, escalating needs of vasoactive support, or worsening end-organ markers (e.g., rising lactate, decreased mixed venous saturation) and clinical factors (e.g., declining physical activity status, NYHA stage progression, intolerance to increases vasoactive support) prompted the utilization of tMCS with Impella 5.5 placement intended as bridge therapy.

Impella care was standardized as follows:

- Image assessment of Impella placement was conducted with a once-a-week trans-thoracic echocardiogram to assess for right heart failure and device positioning within the left ventricle.
- Hemodynamic assessment after Impella placement was performed using pulmonary artery catheters maintained for a minimum of 24 h, with the total duration of placement ultimately determined by the treatment team. Early device removal strategies were favored, given the increased risk of deep venous thrombosis formation and the need to maintain right internal jugular vein patency for post-transplant endomyocardial biopsy access.
- The standard purge solution in this group was heparin-based. Systemic anticoagulant strategies utilized heparin infusion to mitigate the risk of device-related thrombotic events. Bivalirudin was substituted for patients with concern for the development, or prior history, of heparin-induced thrombocytopenia (HIT) or allergy to heparin products. A target prothrombin time (PTT) was maintained between 50 and 70. Daily lactate dehydrogenase was assessed for thrombus formation and hemolysis. Plasma-free hemoglobin was rarely checked due to our institution's 1-week delay in results as a send-out test.

### Cardiogenic shock therapy

Patient care and adjustment of guideline-directed medical therapies in this population were based on the treating physician’s discretion. Diuretic dosing was individualized based on central venous pressure monitoring, physical examination, lab values, and daily weights. Inotrope therapies were guided by daily mixed venous saturation assessment through a peripherally inserted central catheter (PICC) venous blood gas and Fick calculation of cardiac output and index. A weekly discussion of patients listed for heart or heart-kidney transplantation occurred within the multi-specialty selection conference. Patients awaiting organ transplantation supported with tMCS were reviewed for de-escalation, escalation, or alternative support needs.

### Informed consent

Informed consent was waived by the Mayo Clinic IRB (#22004000) due to the retrospective nature of this study. The Mayo Clinic IRB approved a waiver of HIPAA authorization in accordance with applicable HIPAA regulations.

## Results

### Pre-Impella

The median age was 67 (IQR 58–69), with 1 female and 5 male patients. The average BMI was 31 (IQR 29–33). In our cohort, blood groups included O (4), A (1), and B (1). Pre-admission data 1 year prior to admission are summarized in Table 1. The median left ventricular ejection fraction at the time of Impella placement was 18% (IQR 15–23%). Five patients were non-diabetic. The median pre-impella creatinine was 2.1 mg/dL (IQR 1.75–2.3), and the median estimated glomerular filtration rate was 34 mL/min/BSA (34–40), Fig. 4. Median hematocrit at the time of Impella placement was 32% (IQR 32–34). Pre-impella hemodynamics demonstrated a median mean arterial pressure (MAP) of 73 mmHg (IQR 78–86), RA of 12 mmHg (IQR 11–14), PA mean of 38 mmHg (IQR 38–44), and wedge pressure of 30 mmHg (IQR 28–32). Median CVP/Wedge ratio was 0.43 (IQR 0.43–0.57), pulmonary vascular resistance (PVR) of 2.4 WU (IQR 3.4–4.9), Fick cardiac index of 1.45 L/min/m^2^ (IQR 1.64–1.8) while on single or dual vasoactive support (Table 1).

### Post-Impella

**Figure 2:**
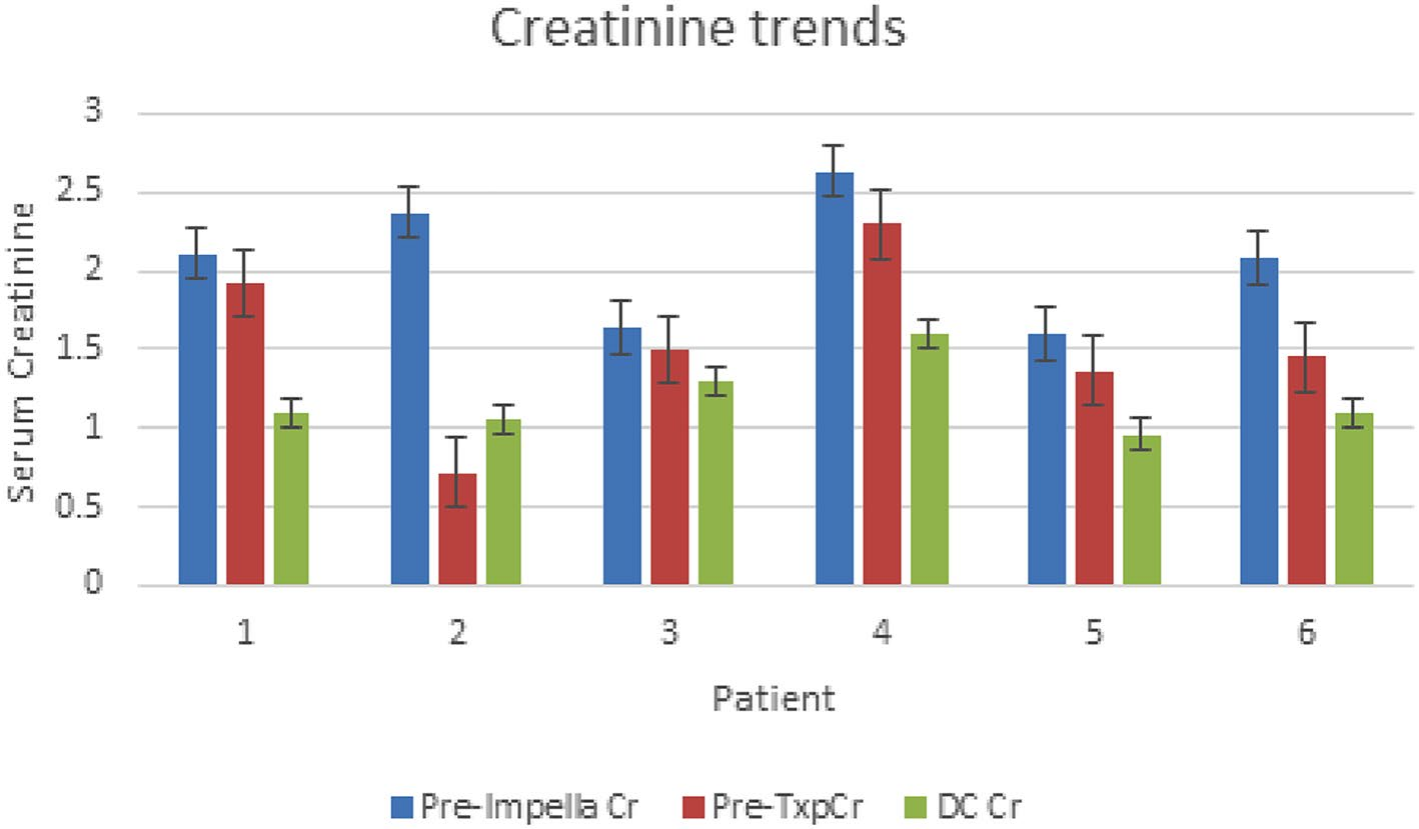
Creatinine trends during hospitalization. Cr = creatinine (g/dL), Pre-Impella Cr = baseline labs; Pre-TxpCr = day before heart transplant labs; DC Cr = labs on day of discharge.

**Figure 3:**
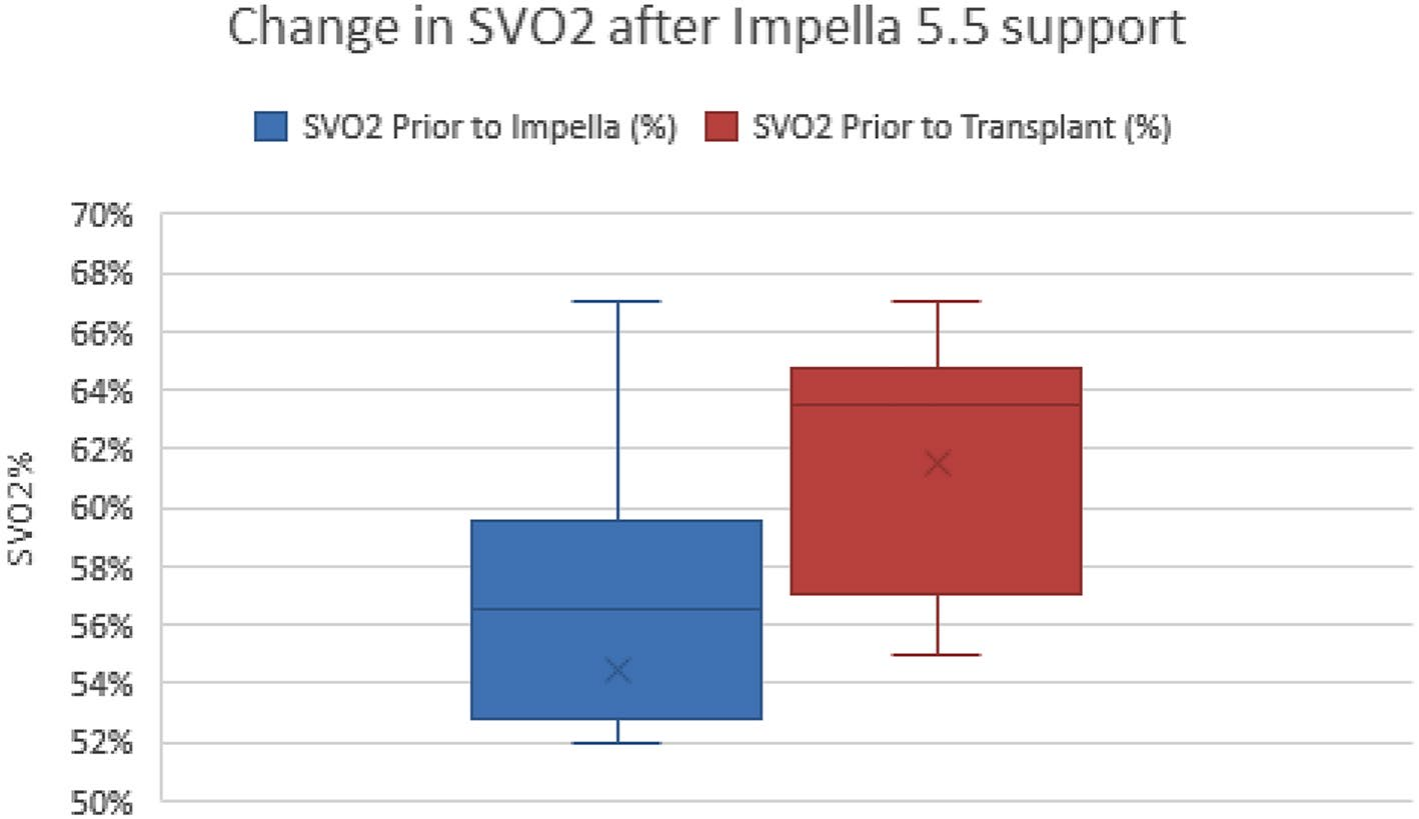
Mixed venous saturation change after Impella. SVO2 = mixed venous saturation (%); Prior to impella = baseline labs; Prior to transplant = day before organ transplant labs.

**Figure 4:**
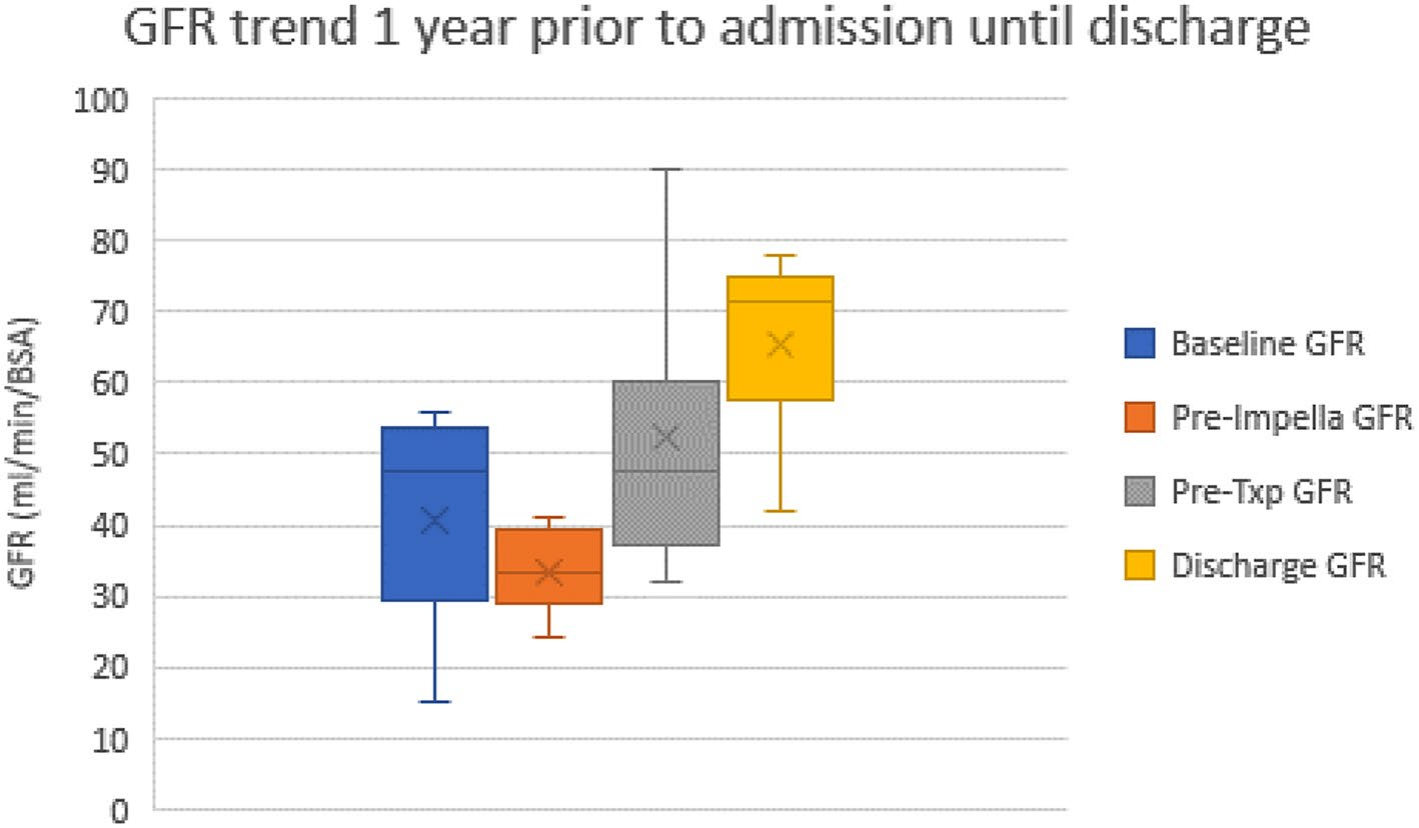
Glomerular filtration rate (GFR) change over time.

**Figure 5:**
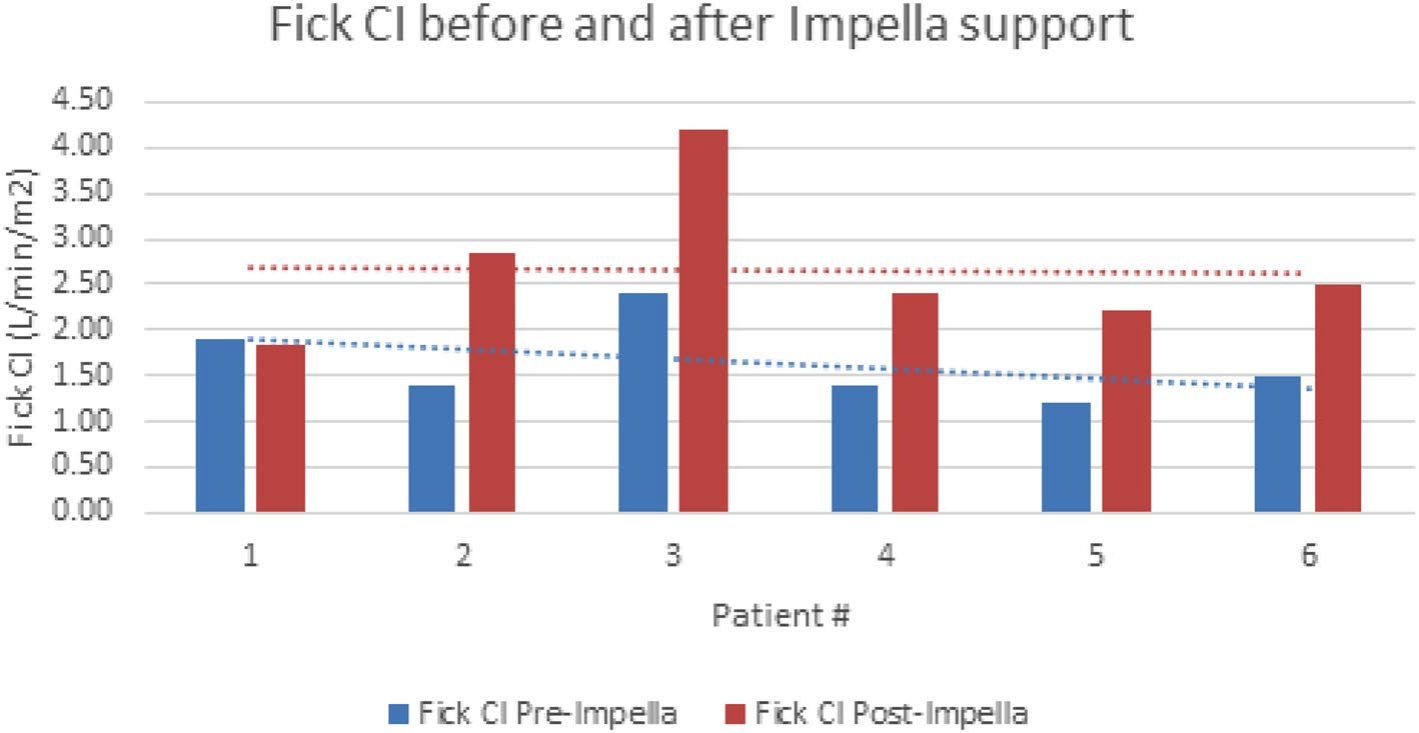
Cardiac index trend during hospitalization. *CI* cardiac index (L/min/m^2^).

The average duration of support with tMCS was 16.5 days before organ transplantation. After placement of the Impella 5.5 device, we observed a trend towards improvement in creatinine, Fick cardiac index, mixed venous saturation, and glomerular filtration rate (GFR), which persisted through transplantation and discharge (Figs. 2, 3, 4, 5). Median GFR improved from 34 to 44 mL/min/BSA (IQR 45–51), with a serum creatinine of 1.5 mg/dL (1.5–1.8) compared to 2.1 mg/dL (2.07–2.3) before Impella placement. Hemodynamics after Impella 5.5 support demonstrated a median MAP of 75 mmHg (IQR 77–78), Fick cardiac output of 5.3 L/min (IQR 5.7–5.8) and cardiac index of 2.45 L/min/m^2^ (IQR 2.6–2.8). Mixed venous saturation of 64% (IQR 62–65) compared to 56% before Impella placement. Cardiac power output, a surrogate of right ventricular function, increased to 0.9 W (pre-impella median of 0.6 W). After Impella placement, median hematocrit was 30% (IQR 32–35), unchanged from the pre-device state. Average lactate dehydrogenase did not indicate concern for hemolysis in any patients.

### Post-transplant

One patient was transplanted as a UNOS status 1 exception due to right ventricular failure, with the other five as UNOS Status 2. The median intraoperative cardiopulmonary bypass time was 162 min (IQR 162–183 min), and the median cold ischemic time was 205 min (IQR 202–224). The median duration of support with Impella was 18 days (IQR 17–26), with a post-transplant ICU length of stay of 5 days. The median hospital length of stay from admission to discharge was 42 days (IQR 38–47). The median discharge creatinine was 1.1 mg/dL (1.19–1.25) with a GFR of 72 (65–74), Fig. 4. None of the six patients required renal replacement therapy after heart transplantation. Most patients had CKD stage 3 pre-device and pre-transplant, which improved to CKD Stage 2 after transplantation.

## Discussion

We present six patients being considered for heart and kidney transplant out of 20 supported with Impella 5.5 for progressive cardiogenic shock as a bridge to transplantation. These six patients demonstrated renal recovery to the point where our multi-disciplinary team was comfortable avoiding renal transplantation. Within all 20 patients reviewed during this timeframe, we found similar trends in renal function improvement (Supplemental Fig. S1). However, we felt it helpful to focus on the data for these six patients being evaluated for heart-kidney transplant that ultimately required heart transplant alone. Renal recovery to this degree is not currently well described in published literature. Below, we outline the currently available literature on cardiorenal syndrome, renal recovery, and Impella 5.5 use. We conclude with a summary of our contribution to the field with data based on our experience.

### Underlying pathophysiology of cardiorenal syndrome

Cardiorenal syndrome involves the dysfunction between the heart and kidneys and the disease progression associated with the interdependence of these organs^10^. Multiple pathophysiological components, including hemodynamic, hormonal, and inflammatory impairments, are involved and ultimately hinder the appropriate function of the heart and kidneys. Increased salt and fluid retention, activation of the renin–angiotensin–aldosterone (RAAS), and sympathetic nervous system result in increased central venous (CVP) and intra-abdominal pressures^11^. Kidney congestion stems from elevated intra-abdominal and CVP, low cardiac output, and tubuloglomerular feedback. The increase in venous pressure compounds inadequate blood flow across the vasculature within the kidneys. As a result of slower flow in the ascending loop of Henle, renin release increases from the juxtaglomerular cells in the afferent arterioles. This results in sodium retention, worse congestion, and decreased urine output. Another factor that contributes to cardiorenal syndrome is oxidative stress. Oxidative stress is the product of an imbalance between oxidants and antioxidants, resulting in the inability of the body to metabolize elevated levels of reactive oxygen species (ROS)^12^. Increased levels of angiotensin II due to the activation of RAAS produces ROS and amplifies oxidative stress in patients with heart failure and kidney disease^13^. Furthermore, increased angiotensin II compounds the stress on the right ventricle and pulmonary vasculature by increasing pulmonary afterload.

### Renal recovery and cardiogenic shock support

There is a lack of evidence surrounding renal recovery in patients with chronic kidney failure in cardiogenic shock supported with tMCS^14^. Additionally, large bodies such as the Acute Kidney Injury Group have worked to guide the clinical and therapeutic definition of renal recovery and broaden the understanding of underlying renal injury and recovery physiology, molecular markers, and predictors of renal recovery with some difficulty^15, 16^.

We do know, however, that intracardiac chamber filling pressures have long been described as critical markers of renal dysfunction and increased mortality^17–19^. Acute decompensated heart failure and the progressive decline to cardiogenic shock with both right and left heart failure and systemic congestion eventually lead to multi-organ dysfunction^20^. The literature surrounding renal disease and outcomes with mechanical circulatory support in heart failure patients focuses on durable LVAD and acute or chronic kidney failure^21^. The Centers for Medicare and Medicaid Services have clearly stated that irreversible renal disease is an absolute contraindication to the placement of durable LVAD. However, a study of Medicare beneficiaries by Walther et al. in 2018 discussed the use of LVAD in patients requiring renal replacement therapy due to a combination of acute and chronic kidney disease^22^. As expected, the survival outcomes of this data were poor, reporting a 1-year mortality of 61.5% in patients undergoing LVAD placement with known renal failure. Older generation tMCS devices such as the Impella CP and 2.5 or even current generation ECMO use are associated with increased hemolysis, directly related to irreversible renal damage from pigment nephropathy, due to a combination of factors: pump size (Impella CP/2.5), pump flow (ECMO), and red blood cell shear stress among all three–independent of systemic anticoagulant usage^15, 23–26^. Our own and other published data show this is rarely seen with the Impella 5.5^27–30^. Furthermore, it is imperative to understand the acute decompensation of heart failure progressing to cardiogenic shock, necessitating escalation to vasoactive support followed by tMCS placement, highlights a population with an acute on chronic renal insult. As we show in Fig. 4, the acute worsening of GFR can be stopped if patients are supported with tMCS, and, compared to published data, remains stable or further improves through organ transplantation^10, 16^.

### Organ transplant equity and the potential for renal recovery

We used Impella 5.5 support to manage cardiogenic shock in patients with a long-standing history of heart failure and stage 3 worse chronic kidney disease. The use of the Impella 5.5 device in this group was based on the inability to offload the left ventricle, the need for escalation of inotrope support, or progressive decline as evidenced by clinical and laboratory data, including worsening kidney function. Patients were evaluated for heart and kidney transplantation based on individual characteristics, which were driven by worsening kidney function despite an escalation in vasoactive support prior to Impella placement. The intent of early escalation to Impella 5.5 is to prevent the onset of cardiometabolic shock and irreversible end-organ damage.

As outlined in our supplemental data, we found multiple patients who experienced optimization in renal function, indicating that Impella support has a stepwise improvement upon cardiac output beyond standard vasoactive support. Furthermore, there is a demonstrated decrease in bi-ventricular filling pressures and a probable effect upon the mitigation of worsening acute neurohormonal surge in shock.

Reconsideration for obviating the need for renal transplantation in these six patients occurred due to marked improvement in GFR and avoidance of initiation of renal replacement therapies. Within our case series, baseline GFR in all of our patients was < 60 mL/min/BSA, with more than half moderate CKD stage 3b or worse. This drove an early team-based approach to consider heart-kidney transplantation due to the risk of worse outcomes with post-cardiopulmonary bypass-related renal injury.

This improvement was demonstrated through improved GFR, preservation of right heart function, and sustained renal improvement without requiring renal replacement therapy before or after heart-only transplantation. These results are a significant improvement over other short or long-term mechanical circulatory support options we outlined. The use of Impella has the potential to improve patient outcomes and lead to sustained renal recovery and stabilization in heart failure cardiogenic shock.

### Post-transplant management

Furthermore, these patients tolerated the standard induction immunotherapy (thymoglobulin or basiliximab) without delaying the initiation of the calcineurin inhibitor, tacrolimus. All patients had initiation of tacrolimus within the first 96 h after extubation. Daily post-transplant complete blood counts were evaluated for potential thrombocytopenia (e.g., platelet count < 50,000). This finding is significant because induction immunotherapy can allow for a safe delay in initiating calcineurin inhibitor therapy to avoid renal injury. The fact that the patients did not experience kidney injury and could tolerate the standard immunotherapy regimen after heart transplantation suggests that their renal function had improved and stabilized despite undergoing the stress of cardiopulmonary bypass and expected vasoplegia after organ transplantation.

### Pluripotent impact of Impella in cardiogenic shock and chronic kidney disease

In our limited case series, we found clinically significant improvement in kidney function in patients awaiting heart transplantation supported with the Impella 5.5 with SmartAssist. Patient-specific data highlights the broader impact of Impella 5.5 support in patients with AKI on CKD and cardiogenic shock—allowing for 1. Optimization of hemodynamic support (e.g., minimizing post-Impella vasoactive needs), 2. Biventricular functional alignment (as evidenced by CPO, SVO2, and CI), and 3. Splanchnic and renal venous decongestion (demonstrated with improved renal function and hemodynamic assessment).

### Limitations

This data is presented as hypothesis-generating. We look to examine a new pathway for renal recovery/stabilization in cardiogenic shock with the support of the Impella device. Factors influencing renal function that are not under full control in the intensive care setting include accurate capture of urine output (patient cooperation, mixed urine/stool output, etc.…) and duration or utilization of invasive catheters for intracardiac filling pressure assessment. During our review of these six patients, urine output remained unchanged before or after Impella use. We also worked to quantify total diuretic dose and albumin/colloid blousing to balance the effect of competing factors in modifying renal function—however, no trends were highlighted. This may be due to practice variations amongst critical care or nephrology providers at different times during patient care. Overall, however, the general dosing of diuretics was 2.5 mg IV once or twice daily of bumetanide with intermittent use of 250 or 500 mg of IV Chlorothiazide before and after Impella support.

## Conclusion

Impella 5.5 use in our patient population resulted in renal recovery across all patients, with a further improvement in six patients who had significant renal dysfunction but eventually obviated the need for renal transplantation. Given the minimally invasive placement via axillary cutdown, limited hemolysis profile, and biventricular offloading—the Impella 5.5 with SmartAssist presents a new paradigm in optimization for patients with advanced kidney disease awaiting organ transplantation in cardiogenic shock. Further studies with larger cohorts should be considered.

### Supplementary Information

Supplementary Figure 1.

## Data Availability

All data are available upon reasonable request by the corresponding author.
