# Supplementary material for: Axillary mechanical circulatory support improves renal function prior to heart transplantation in patients with chronic kidney disease
Source: Sci Rep. 2023 Nov 11;13:19671. doi: 10.1038/s41598-023-46901-7 (PMC10640571; doi:10.1038/s41598-023-46901-7)
Supplement: Supplementary file 1 — Supplementary Figure 1. [file 41598_2023_46901_MOESM1_ESM.docx]

Supplemental Figure 1: GFR Trend for patients with Impella 5.5 during transplant episode of care


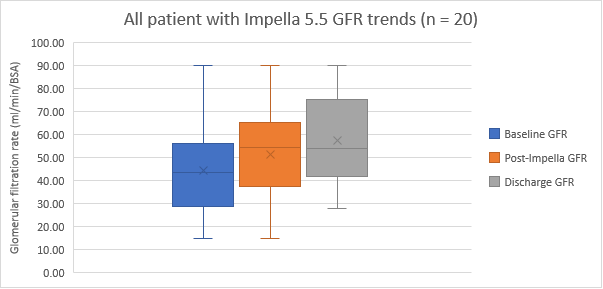


GFR = glomerular filtration rate (ml/min/BSA); Baseline: admission day lab value; post-impella: day before heart transplantation lab value; discharge: day of discharge lab value.
